# Circulating Cell-Free DNA Assessment in Biofluids from Children with Neuroblastoma Demonstrates Feasibility and Potential for Minimally Invasive Molecular Diagnostics

**DOI:** 10.3390/cancers14092080

**Published:** 2022-04-21

**Authors:** Marco Lodrini, Jasmin Wünschel, Theresa M. Thole-Kliesch, Maddalena Grimaldi, Annika Sprüssel, Rasmus B. Linke, Jan F. Hollander, Daniela Tiburtius, Annette Künkele, Johannes H. Schulte, Erwin Lankes, Thomas Elgeti, Patrick Hundsdörfer, Kathy Astrahantseff, Thorsten Simon, Angelika Eggert, Hedwig E. Deubzer

**Affiliations:** 1 Department of Pediatric Hematology and Oncology, Charité—Universitätsmedizin Berlin, 13353 Berlin, Germany; marco.lodrini@charite.de (M.L.); jasmin.wuenschel@charite.de (J.W.); theresa.thole@charite.de (T.M.T.-K.); grimaldi.madda@gmail.com (M.G.); annika.spruessel@gmx.de (A.S.); rasmus.linke@charite.de (R.B.L.); jan-fredrik.hollander@charite.de (J.F.H.); d.tiburtius@web.de (D.T.); annette.kuenkele@charite.de (A.K.); johannes.schulte@charite.de (J.H.S.); patrick.hundsdoerfer@helios-gesundheit.de (P.H.); kathy.astrahantseff@charite.de (K.A.); angelika.eggert@charite.de (A.E.); 2 Experimental and Clinical Research Center (ECRC) of the Charité and the Max-Delbrück-Center for Molecular Medicine (MDC) in the Helmholtz Association, 13125 Berlin, Germany; 3 Max-Delbrück-Center for Molecular Medicine (MDC) in the Helmholtz Association, 13125 Berlin, Germany; 4 German Cancer Consortium (DKTK), Partner Site Berlin, 10117 Berlin, Germany; 5 German Cancer Research Center (DKFZ), 69120 Heidelberg, Germany; 6 Berlin Institute of Health at Charité—Universitätsmedizin Berlin, Charitéplatz 1, 10117 Berlin, Germany; 7 Department of Pediatric Endocrinology and Diabetes, Charité—Universitätsmedizin Berlin, 13353 Berlin, Germany; erwin.lankes@charite.de; 8 Center for Chronically Sick Children, Charité—Universitätsmedizin Berlin, 13353 Berlin, Germany; 9 Department of Radiology (including Pediatric Radiology), Charité—Universitätsmedizin Berlin, 13353 Berlin, Germany; thomas.elgeti@charite.de; 10 Department of Pediatric Oncology, Helios Klinikum Berlin Burch, 13125 Berlin, Germany; 11 Department of Pediatric Oncology and Hematology, Children’s Hospital, University of Cologne, 50924 Cologne, Germany; thorsten.simon@uk-koeln.de

**Keywords:** liquid biopsy, cancer, detection of therapeutic targets, minimal residual disease, precision medicine, real-time monitoring of therapeutic efficacy, *ALK* mutation, ALK-inhibitor, *MYCN* amplification

## Abstract

**Simple Summary:**

The invasive nature of surgical biopsies prevents their sequential application to monitor disease. Single biopsies fail to reflect cancer dynamics, intratumor heterogeneity, and drug sensitivities that change over time. Detection and characterization of cell-free circulating tumor DNA in biofluids from patients with solid tumors may better support disease monitoring and provide advanced molecular information for clinical decision-making toward personalized medicine. Here, we investigated the cell-free DNA characteristics in blood, bone marrow, cerebrospinal fluid, and urine provided from 84 infants and children with low-, intermediate-, or high-risk neuroblastoma. We report characteristic size distribution and concentration patterns for each biofluid to provide information to support the development of successful liquid biopsy biobanking strategies. We investigate potential correlations between disease activity and cfDNA concentration and provide strong evidence that markers specific for neuroblastoma can be detected in very small blood volumes from infants.

**Abstract:**

Liquid biopsy strategies in pediatric patients are challenging due to low body weight. This study investigated cfDNA size distribution and concentration in blood, bone marrow, cerebrospinal fluid, and urine from 84 patients with neuroblastoma classified as low (*n* = 28), intermediate (*n* = 6), or high risk (*n* = 50) to provide key data for liquid biopsy biobanking strategies. The average volume of blood and bone marrow plasma provided ranged between 1 and 2 mL. Analysis of 637 DNA electropherograms obtained by Agilent TapeStation measurement revealed five different major profiles and characteristic DNA size distribution patterns for each of the biofluids. The proportion of samples containing primarily cfDNA was, at 85.5%, the highest for blood plasma. The median cfDNA concentration amounted to 6.28 ng/mL (blood plasma), 58.2 ng/mL (bone marrow plasma), 0.08 ng/mL (cerebrospinal fluid), and 0.49 ng/mL (urine) in samples. Meta-analysis of the dataset demonstrated that multiple cfDNA-based assays employing the same biofluid sample optimally require sampling volumes of 1 mL for blood and bone marrow plasma, 2 mL for cerebrospinal fluid, and as large as possible for urine samples. A favorable response to treatment was associated with a rapid decrease in blood-based cfDNA concentration in patients with high-risk neuroblastoma. Blood-based cfDNA concentration was not sufficient as a single parameter to indicate high-risk disease recurrence. We provide proof of concept that monitoring neuroblastoma-specific markers in very small blood volumes from infants is feasible.

## 1. Introduction

The embryonal tumor, neuroblastoma, accounts for up to 15% of all cancer-related deaths in children [1]. Its heterogeneous tumor biology creates clinical variability spanning from spontaneous regression to therapy-refractory metastasizing progression [2]. Despite considerable international efforts to improve outcomes, long-term survival of the high-risk disease remain poor, with <50% overall survival after first-line treatment and <10% after relapse [3,4]. Liquid biopsy-based diagnostic approaches may have the power to sustainably improve clinical care for children with neuroblastoma by reflecting precise disease status and detecting druggable targets at any time during treatment and follow-up. The invasive nature of surgical biopsies most often prevents their sequential application to monitor disease. Single biopsies also fail to reflect cancer dynamics, intratumor heterogeneity among micro- and macrometastases, and drug sensitivities that most likely change during clonal evolution and under the selective pressure of therapy [5,6]. Liquid biopsy components have a wide range of attractive clinical applications, including prognostication at diagnosis, treatment response assessment, early metastasis detection, and targeted drug selection, allowing a personalized treatment choice [5,6]. Emerging data indicate that implementing molecular characterization of tumor surrogates (cell-free nucleic acids, circulating tumor cells, extracellular vesicles) over time will improve outcome prediction, patient monitoring, and treatment selection for cancer patients as well as capture the molecular landscape of all tumor clones, follow clonal evolution in tumor subpopulations and provide a basis for clinical decision-making by closely measuring treatment response [5,6].

Individual assays employing circulating cell-free DNA (cfDNA) have already been incorporated into routine disease monitoring for cancer entities afflicting adult patients. To date, no individual assays have been approved for standard-of-care routine use in pediatric oncology, indicating that the pediatric field lags behind its adult counterpart in spite of an increasing number of small retrospective proof-of-concept studies [5,6]. At least 30 studies have reported cfDNA findings in patients with neuroblastoma. A total of 15 studies investigated copy-number variations [7,8,9,10,11,12,13,14,15,16,17,18,19,20,21], 6 single-nucleotide variations and indels [16,17,20,21,22,23], 5 translocations or rearrangements [13,24,25,26,27], 5 total cfDNA levels [15,21,28,29,30], 4 DNA methylation [31,32,33,34], 2 segmental chromosomal alterations [35,36], and 1 clonality [16]. Multiple assays employing cfDNA as input material were applied in nine of these studies [13,15,16,17,20,21,25,26,27]. Collectively, these studies demonstrate that patients with neuroblastoma have blood cell-free circulating tumor DNA levels that are detectable with next-generation sequencing and PCR-based approaches [7,8,9,10,11,12,13,14,15,16,17,18,19,20,21,22,23,24,25,26,27,28,29,30,31,32,33,34,35,36]. The low amounts of cfDNA in liquid biopsies from pediatric patients, however, continue to make these testing strategies challenging for patients with childhood cancers including neuroblastoma. Validation of findings from small proof-of-concept studies is warranted in large prospective trials. To provide the indispensable technical basis to support liquid biopsy biobanking for pediatric patients, we investigated cfDNA size distribution and total cfDNA levels in peripheral blood, bone marrow, cerebrospinal fluid (CSF), and urine from neuroblastoma patients collected under standardized pre-analytical sample handling conditions [37]. Cell-free DNA levels were correlated with disease activity and the feasibility to detect neuroblastoma-specific markers in the particularly small blood sample volumes from infants was tested.

## 2. Materials and Methods

### 2.1. Patient Samples

**Figure 1:**
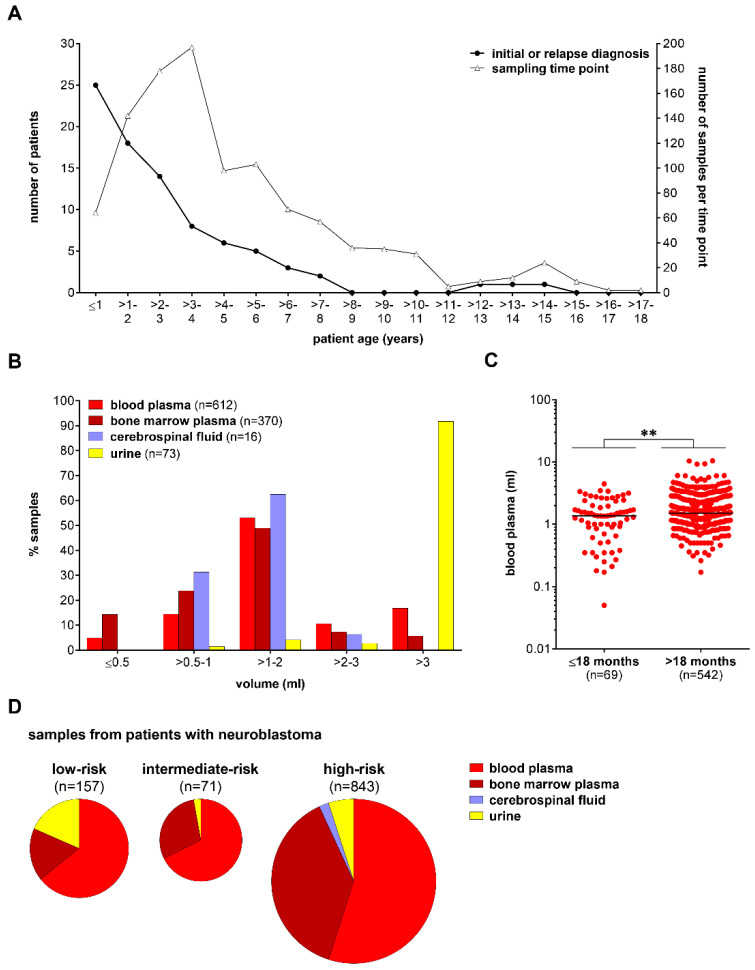
Overview of liquid biosamples from patients with neuroblastoma. (**A**) Line chart summarizing patient age at diagnosis and patient age at individual sample time point for the sequentially collected biofluids. (**B**) Bar chart indicating the percentage of biofluids according to sample volume. (**C**) Dot chart demonstrating blood plasma volumes provided from patients ≤ and >18 months of age. ** *p* < 0.01. (**D**) Multiple pie charts visualizing the distribution of biosamples collected in this study according to treatment stratification of patients with neuroblastic tumors [38].

Blood and bone marrow plasma, CSF, and urine samples were collected (local ethics approval: EA2/055/17) from a total of 84 patients with neuroblastoma classified as low (*n* = 28), intermediate (*n* = 6), or high (*n* = 50) risk by the German Neuroblastoma Risk Classification System [38]. The median patient age in the overall study population was 23.2 months (min–max: 1 day–169 months). The median patient age was 6.3 months for the low-risk neuroblastoma subgroup (min–max: 1 day–162 months), 14.9 months for the intermediate-risk subgroup (min–max: 0.8–70.7 months), and 31.7 months for the high-risk subgroup (0.6–169 months, patient/tumor characteristics summarized in Supplementary Table S1). Altogether, 90.5% of patients with neuroblastoma were up to six years of age at the time of first sample collection, and 73% of longitudinally collected samples belonged to this age group (Figure 1A). Patients were treated at the Charité—Universitätsmedizin Medizin Berlin and registered either in the German NB2004 Trial (EudraCT 20661) or the NB 2016 Registry. Informed written patient/parent consent was obtained during trial/registry participation. Blood plasma was likewise collected (local ethics approval: EA2/131/11) from 25 pediatric patients with non-malignant conditions and a median age of 72.4 months (min–max: 21.6–244.4 months) as comparative control samples [21]. Bone marrow plasma was collected (local ethics approval: EA4/132/17) from 43 healthy individuals with a median age of 26.3 years (min–max: 4.5–46.4 years), who were acting as bone marrow donors.

Peripheral blood and bone marrow were uniformly collected in EDTA tubes without the addition of stabilizers while CSF and urine were collected in sterile polypropylene screw-cap tubes [21]. The average time from biosample collection to laboratory entrance was 1.0 h for blood (interquartile range 0.5–2.4 h), 0.9 h for bone marrow (interquartile range 0.5–1.5 h), 1.0 h for CSF (interquartile range 0.5–1.6 h), and 1.3 h for urine (interquartile range 0.8–2.1 h). In total, 80.8% of blood samples, 89.2% of bone marrow samples, 93.7% of CSF samples, and 75.3% of urine samples were processed within the recommended time interval of four hours [37]. Blood samples that could not be processed within four hours due to delayed laboratory entry contained an overall higher amount of cfDNA, which reached statistical significance (*p* < 0.05) (Supplementary Figure S1A). Further, the proportion of samples containing primarily gDNA was higher (Supplementary Figure S1B). This study did not focus on determining the circulating cell-free tumor DNA fraction in the overall circulating cell-free DNA content. One may speculate that the higher overall cfDNA content measured in blood plasma that was processed with delay may be due to DNA degradation affecting all DNA molecules independent of its tumor versus non-tumor origin.

Peripheral blood, CSF, and urine were centrifuged at 1900× *g* for 7 min to separate plasma or remove cell debris [21]. Bone marrow was centrifuged at 450× *g* for 7 min to separate plasma from cells [21]. All samples were centrifuged a second time at 3250× *g* for 10 min before storage at −80 °C [21]. The average volume of blood plasma, bone marrow plasma, and CSF from patients with neuroblastoma ranged between 1 and 2 mL (Figure 1B). In total, less than 4.9% of blood plasma and 14.3% of bone marrow plasma samples had a volume ≤0.5 mL (Figure 1B). The median blood plasma volume provided from patients with neuroblastoma below the age of 18 months was significantly lower compared to those available from older patients (Figure 1C). The predominant biosample provided from patients with neuroblastoma was peripheral blood (*n* = 612), reaching 64.3% in the low-risk subgroup, 67.6% in the intermediate-risk subgroup, and 54.9% in the high-risk subgroup (Figure 1D). The proportion of bone marrow plasma samples available for molecular diagnostics (total number *n* = 370) was, at 38.2%, the highest in the high-risk subgroup (Figure 1D). CSF samples (*n* = 16) were exclusively available from patients with isolated central nervous system relapses and Rickham reservoirs required for repeated intrathecal chemotherapeutic drug delivery (Figure 1D).

### 2.2. Genomic and Cell-Free DNA Preparation

Genomic DNA was extracted from tumor tissue using the Qiagen Puregene Core kit A (Qiagen) or the QIAamp DNA Mini kit (Qiagen) according to the manufacturer’s instructions and quantified on a Qubit 2.0 fluorometer (Life Technologies). Fragmentation of genomic DNA was achieved by 5 U of AluI or HaeIII restriction enzyme (New England Biolabs) added to each droplet digital PCR (ddPCR) reaction [14]. Thawed blood and bone marrow plasma, CSF, and urine samples were centrifuged at 2000× *g* for 5 min to clear debris, then supernatants were centrifuged at 20,000× *g* for 5 min. Cell-free DNA was purified from a minimum of 40 µL stored samples using the QIAamp Circulating Nucleic Acid kit (Qiagen), then concentrated to 50 μL using the DNA Clean and Concentrator-5 kit (Zymo Research), both according to manufacturers’ directions. Total cfDNA was quantified using the cfDNA ScreenTape assay (Agilent) and Agilent 4200 TapeStation System according to the manufacturer’s instructions [17]. DNA size distribution was likewise assessed with the Agilent 4200 TapeStation System [17]. DNA fragments between 100 and 300 bp were considered to be total cfDNA [39]. Assessment of DNA size distribution and cfDNA quantity does not allow any conclusion to be drawn about the cell(s) of origin.

### 2.3. Droplet Digital PCR

The QX200 Droplet Digital PCR System (Bio-Rad) was used to quantify *MYCN* (2p24.3) and *ALK* (2p23.2-2p23.1) copy number and screen for *ALK* p.F1174L (3522, C>A) and *ALK* p.R1275Q (3824, G>A) hotspot mutations with their corresponding wildtype sequences in ddPCR assays [14,17,22]. The following T100 Thermo Cycler (Bio-Rad) programs were performed: (i) copy-number variation: denaturation at 95 °C for 10 min, 40 cycles of 30 s at 94 °C and 1 min at 58 °C and final denaturation for 10 min 98 °C and (ii) single-nucleotide variation: denaturation at 95 °C for 10 min, 40 cycles of 30 s at 94 °C and 1 min at 62.5 °C and final denaturation for 10 min at 98 °C. Target gene copy-number variation and mutant allele frequency (MAF) were analyzed using QuantaSoft Analysis software, version 1.7.4.0917 (Bio-Rad). All assays contained appropriate non-template, positive and negative controls in each run to enable the software to generate specific thresholds. Amplification of the *MYCN* or *ALK* gene was defined as detecting ≥ 8.01 gene copies by ddPCR analysis, while 2.74 to 8.00 copies indicated a gene gain and 1.50 to 2.73 copies indicated the normal diploid gene contingent [14]. In the background of plasma, 1 ng circulating cell-free tumor DNA is required to reliably quantify tumor-specific copy-number alterations [21]. The false-positive rate and limit of detection for single-nucleotide variant analyses were calculated as described [17] using Bio-Rad lookup tables in line with the model by Armbruster and Pry [40].

### 2.4. Statistical Analysis

The non-parametric Mann–Whitney U test evaluated the significance of differences between total cfDNA concentrations in biofluids from patients with neuroblastoma and pediatric patients with non-malignant conditions (blood, urine) or healthy individuals (bone marrow). All tests were conducted using GraphPad Prism version 7.0 (GraphPad Software, San Diego, CA, USA) and *p*-values below 0.05 were considered significant.

## 3. Results

### 3.1. Cell-Free DNA Content Varies among Different Biofluids from Patients

**Figure 2:**
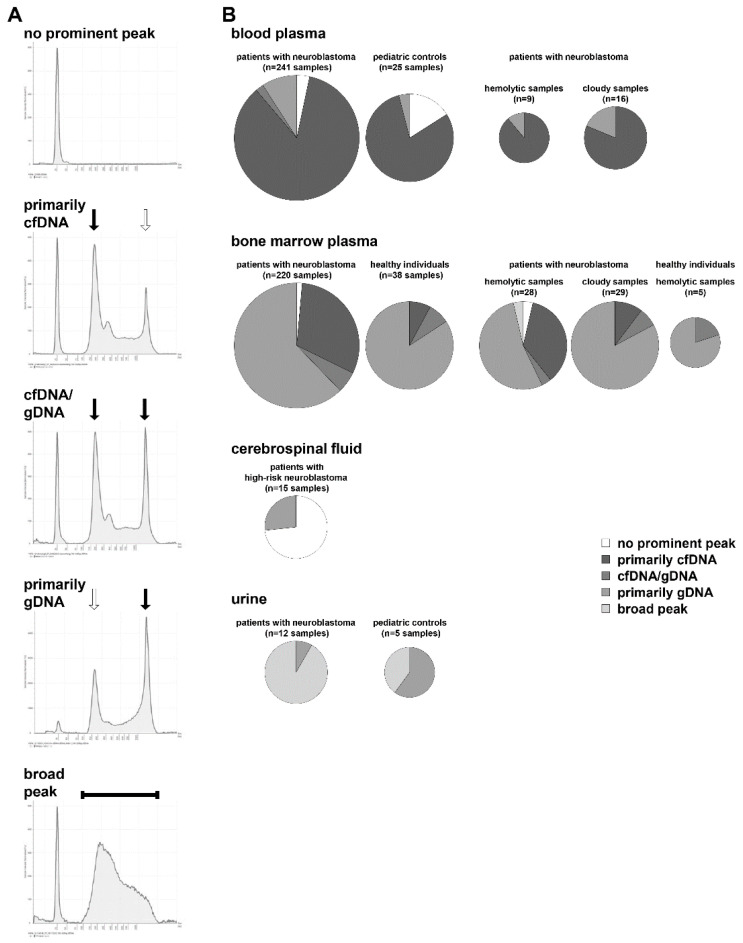
Analytical specification of cell-free DNA in biofluids. (**A**) DNA extracted from biofluids was analyzed using the cell-free DNA ScreenTape assay (Agilent) and the Agilent 4200 TapeStation System. Shown are five electropherogram profiles of cfDNA samples that represent differing sample quality dependent on the amount of high molecular weight material detected and the identification of the typical peak at approximately 170 bp representing the mononucleosome. Vertical arrows indicate peaks characteristic for the cfDNA fraction and/or the high molecular weight genomic DNA. The predominant DNA fraction is highlighted with a black arrow, whereas the smaller DNA fraction is marked with a white arrow. The broad peak characteristic in some electropherograms is indicated with a solid black horizontal line. (**B**) Multiple pie charts demonstrating the distribution of electropherogram profiles characterized by (i) no prominent peak, (ii) primarily cfDNA, (iii) similar amounts of cfDNA and high molecular weight genomic DNA, (iv) primarily high molecular weight genomic DNA and (v) a dominant broad peak among all blood plasma, bone marrow plasma, cerebrospinal fluid and urine samples collected from patients with neuroblastoma, pediatric controls and healthy individuals.

Sample volume, transport, blood collection tubes, and storage conditions sustainably influence cfDNA yield and purity for subsequent analyses [37]. To obtain a representative overview of sample characteristics, we studied electropherogram profiles of cfDNA purified from blood, bone marrow, CSF, and urine obtained from patients with neuroblastoma according to standard operating procedures with strict adherence to time and temperature limits [14,17,21]. In total, 241 blood-based cfDNA samples from patients with neuroblastoma were analyzed on an Agilent 4200 TapeStation. Altogether 9 blood plasma samples showed evidence of hemolysis and 16 plasma samples appeared cloudy. As comparative controls, we analyzed 25 blood-based cfDNA samples from pediatric patients with non-malignant conditions. Cell-free DNA purified from 220 bone marrow plasma samples was available from patients with neuroblastoma (28 hemolytic, 29 cloudy) and from 38 healthy individuals (5 hemolytic, 0 cloudy). A total of 15 CSF-based cfDNA samples from patients with high-risk neuroblastoma were analyzed, as were urine-based cfDNA samples from patients with neuroblastoma (*n* = 12) and pediatric controls (*n* = 5). All of the 637 electropherograms fell into one of five major profiles: (i) no prominent peak detected, (ii) primarily cfDNA detected, (iii) approximately equal amounts of cfDNA and high molecular weight genomic DNA detected, (iv) primarily genomic DNA detected, and (v) broad peak displayed which includes the size range of cfDNA (Figure 2A). 

In total, 85.5% of samples purified from blood plasma primarily contained cfDNA independent of evidence for hemolysis or cloudy appearance (Figure 2B). Despite the strict adherence to standard operating procedures, 2.1% of samples contained approximately equal amounts of cfDNA and genomic DNA, and 9.1% of samples contained primarily genomic DNA. The sample proportion without a prominent peak (due to very low DNA content) reached 3.3% in the patient subgroup and 16% in the pediatric control subgroup (Figure 2B). DNA purified from bone marrow plasma consisted primarily of genomic DNA in 62.3% of samples from patients with neuroblastoma and in 84.2% of samples from healthy individuals (Figure 2B). The DNA size distribution from hemolytic or cloudy bone marrow plasma samples did not substantially differ (Figure 2B). Altogether, only 30.9% of bone marrow plasma samples from neuroblastoma patients and 7.9% of samples from healthy individuals contained primarily cfDNA (Figure 2B). The DNA yield from CSF was too low to detect a prominent peak in 73.3% of samples, with the remaining 26.7% of samples containing primarily genomic DNA (Figure 2B). Urine-based DNA electropherograms were characterized by a broad peak (91.7% of samples from patients with neuroblastoma, 40% of samples from pediatric controls) or peaks indicative of primarily genomic DNA (in the remaining 8.3% and 60% of respective samples, Figure 2B). Our data demonstrate that different DNA size distribution patterns occur in the four analyzed biofluids independent of disease status. The strongest differences in DNA size distribution patterns between patients with neuroblastoma and controls were observed in bone marrow plasma and urine samples.

### 3.2. Multiple cfDNA Assays Can Be Conducted from 1 mL Collected Blood or Bone Marrow Plasma

**Figure 3:**
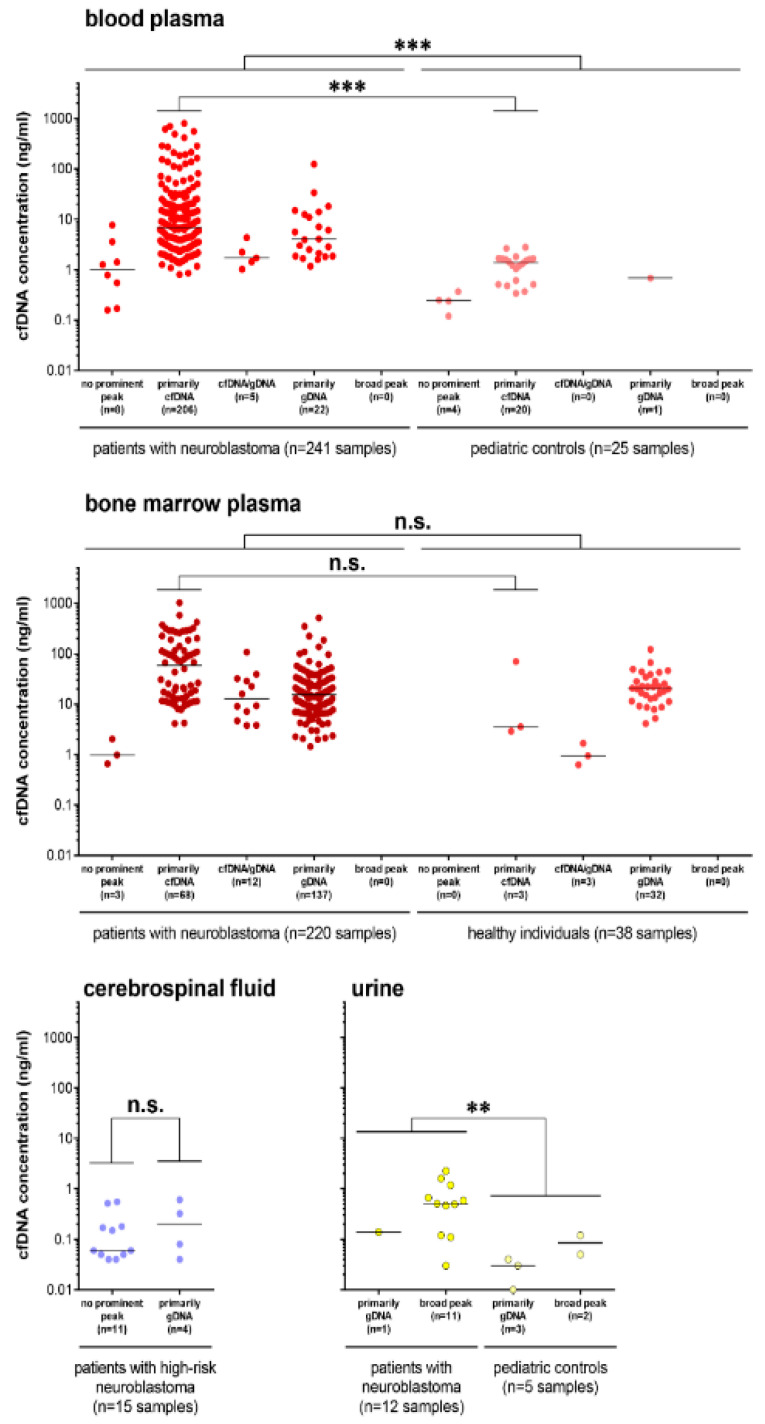
Summary of total cell-free DNA concentrations in biofluids from patients with neuroblastoma and controls. Shown are cfDNA concentrations in blood plasma, bone marrow plasma, cerebrospinal fluid and urine from patients with neuroblastoma and, where applicable, from pediatric controls or healthy individuals. Each symbol depicts an individual measurement. Data are categorized into five different subgroups according to their specific DNA electropherogram. n.s., not significant; ** *p* ≤ 0.01; *** *p* ≤ 0.001.

To establish lower limits for sample volumes required for successful subsequent cfDNA analyses, we quantified cfDNA extracted from biofluids from patients with neuroblastoma and pediatric controls. The blood-based cfDNA content was 5.2-fold higher in patients with neuroblastoma (median concentration = 6.28 ng/mL) than pediatric controls (Figure 3). This analysis included all samples from patients with neuroblastoma, independent of risk group, sampling time point, and DNA profile. Excluding samples with suboptimal electropherogram profiles (only profiles primarily detecting cfDNA) recapitulated the 5-fold higher cfDNA concentration in samples from patients with neuroblastoma compared to pediatric controls (Figure 3). A comparison of bone marrow plasma samples containing a profile detecting primarily cfDNA content revealed a 16.3-fold higher cfDNA concentration in patients with neuroblastoma compared to healthy controls, independent of risk group, sampling time point, and bone marrow involvement (Figure 3). The CSF harbored the lowest cfDNA concentration (median = 0.08 ng/mL) of any of the four analyzed biofluids (Figure 3). Importantly, samples without prominent peaks in their electropherogram profile did contain quantifiable levels of cfDNA with a median of 0.06 ng/mL (Figure 3). The median urine-based cfDNA concentration from patients with neuroblastoma was, at 0.5 ng/mL, 12.4-fold higher than cfDNA measured in urine from pediatric controls. Based on this representative data set for the DNA size distribution and cfDNA content in four biofluids from patients with neuroblastoma, we performed a meta-analysis to define the lower limits of volume size required for cfDNA diagnostics.

**Figure 4:**
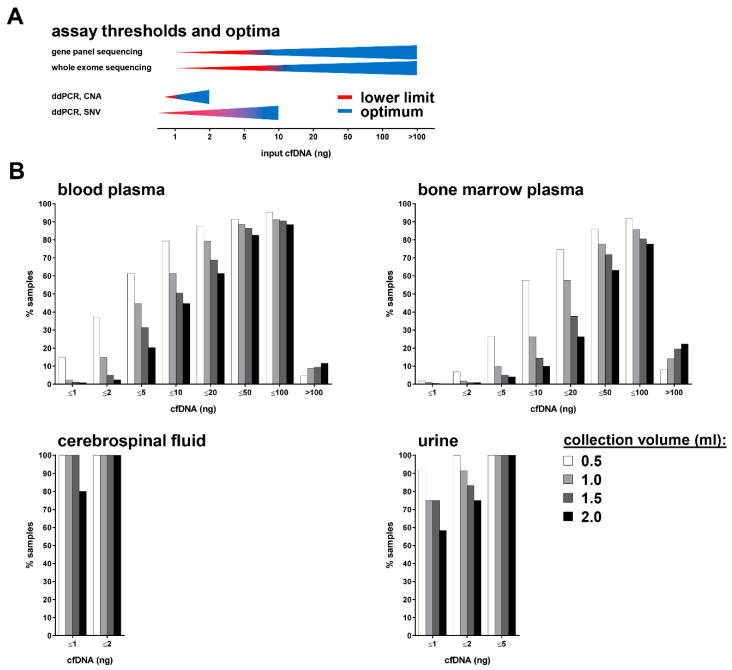
Comparison of cell-free DNA availability with minimum and optimum input requirements for cfDNA analysis. (**A**) Schematic diagram summarizing the sample requirements of selected sequencing and PCR technologies [16,41,42,43]. (**B**) Percentage of samples fulfilling the DNA input thresholds as indicated. CNA, copy number alteration; SNV, single nucleotide variation.

The lower limits for gene panel sequencing and whole-exome sequencing using cfDNA as input are considered to be 5 and 10 ng, respectively (Figure 4A) [16,41,42,43]. Assessing copy-number alterations and single-nucleotide variations using ddPCR requires a minimum of 1 and 5 ng cfDNA, respectively, as input material (Figure 4A) [21]. Our data set indicates that 79.3% of the 0.5 mL blood plasma samples, 61.4% of the 1.0 mL samples, 50.6% of the 1.5 mL samples, and 44.8% of the 2 mL samples contained ≤ 10 ng cfDNA (Figure 4A). Accordingly, 79.3% of blood plasma samples with a maximal volume of 0.5 mL are completely consumed for a single whole-exome sequencing run (Figure 4B). For the analysis of cfDNA purified from bone marrow plasma, 57.7% of the 0.5 mL samples, 26.4% of the 1.0 mL samples, 14.5% of the 1.5 mL samples, and 10% of the 2.0 mL samples contained ≤ 10 ng cfDNA (Figure 4B). Due to the very low cfDNA content in CSF, 100% of the 0.5 mL, 1.0 mL, and 1.5 mL samples contained ≤ 1 ng cfDNA, demonstrating major restrictions for PCR and sequencing approaches using CSF (Figure 4B). Molecular analyses using cfDNA derived from urine face a similar problem, with 91.7% of the 0.5 mL urine samples, 75% of both the 1.0 mL and the 1.5 mL samples, and 58.3% of the 2.0 mL samples containing ≤ 1 ng cfDNA (Figure 4B). At least for urine, the low cfDNA concentration is potentially solvable by collecting large urine volumes. Our analyses indicate that multiple cfDNA-based assays employing the same biofluid sample will optimally require sampling volumes of 1 mL for blood and bone marrow plasma and ideally 2 mL of CSF.

### 3.3. Favorable Treatment Response in Patients with High-Risk Neuroblastoma Is Associated with a Rapid Decrease in Peripheral Blood-Derived cfDNA

**Figure 5:**
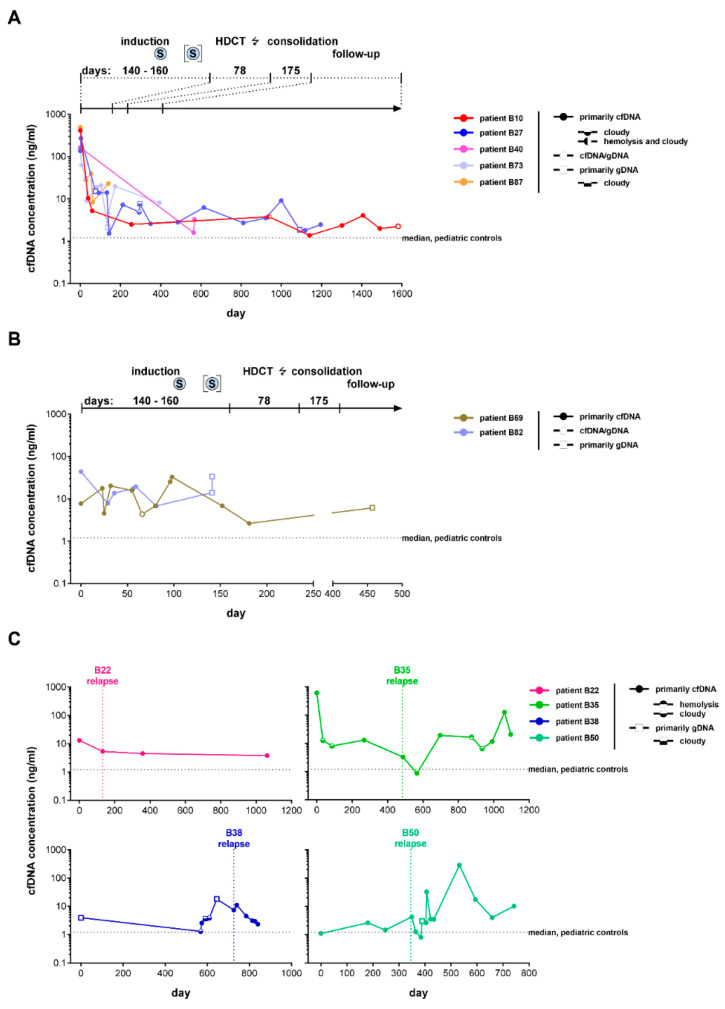
Longitudinal monitoring of cfDNA concentration in blood plasma from patients with high-risk neuroblastoma demonstrates highly variable baseline cfDNA concentrations and rapid cfDNA clearance following favorable treatment response. Total blood-based cfDNA concentrations from patients with a high cfDNA baseline concentration (>50 ng/mL plasma) are summarized in (**A**) while those from patients with a low baseline cfDNA concentration (≤50 ng/mL) are shown in (**B**). Retrospective cfDNA quantification in sequentially collected blood samples from patients with relapsed high-risk neuroblastoma is shown in (**C**). Total cfDNA concentrations were quantified using the Agilent 4200 TapeStation System. Dashed lines indicate the median cfDNA concentration in the pediatric control cohort. The treatment modules according to the German NB2004 Trial (EudraCT 20661) and the consecutive 2017 Guidelines for Diagnosis and Treatment of Patients with Neuroblastic Tumors are indicated above the plots. HDCT, high-dose chemotherapy; ↯, radiation; S, surgery.

Elevated cfDNA levels in the blood of cancer patients were first described by Leon et al. in 1977 [44]. More recently, this finding from the seventies was confirmed for blood from patients with high-risk neuroblastoma [20,21,28]. Su et al. demonstrated dynamic changes in blood-based cfDNA concentrations in response to therapy in patients with high-risk neuroblastoma [29]. We set out to validate this finding by longitudinal assessment of blood-based cfDNA concentration in seven patients with high-risk neuroblastoma prior to, during, and after first-line therapy. Patients B10, B27, B40, B69, B73, B82, and B87 were uniformly treated for high-risk disease according to the German NB2004 Trial or the NB2016 Registry. Therapy response assessment according to RECIST evaluation criteria for solid tumors [45] documented a first complete remission directly following induction therapy in all seven patients. Retrospective cfDNA quantification in samples from patients B10, B27, B40, B73, and B87 documented that cfDNA ranged between 136.0 ng/mL and 492.1 ng/mL at initial diagnosis and dropped below 50 ng/mL directly following induction therapy (Figure 5A). Despite undulations, levels remained low and resembled those detected in samples from the pediatric control cohort (Figure 5A). Patients B69 and B82 initially presented with blood-based cfDNA levels below 50 ng/mL, and no further decrease in cfDNA was observed in response to therapy in these two patients (Figure 5B). Further, there was no correlation between the initial lower level of cfDNA and molecular or clinical characteristics of patients B69 and B82 compared to those of patients B10, B27, B40, B69, B73, B82, and B87 (Figure 5A,B).

Sue et al. reported that a high blood-based cfDNA concentration is a potential molecular marker for high-risk neuroblastoma recurrence [30]. We turned to the longitudinal assessment of cfDNA concentration in surplus biomaterial samples from patients B22, B35, B38, and B50 to test this observation in our study cohort. Patient B22 was diagnosed with a first local relapse in the primary tumor bed during routine follow-up. The overall blood-based cfDNA concentration at this time point was 2.5-fold lower compared to the previous time point (Figure 5C, upper left panel). The technologies applied in this study do not discriminate between circulating cell-free tumor DNA and circulating cell-free DNA released from the various organs in the human body. It is conceivable that the higher cfDNA concentration prior to relapse diagnosis was due to physical exercise, a systemic inflammatory response syndrome, or infection [5]. These conditions are well known to trigger an increased release of cfDNA into the bloodstream through necrosis and apoptosis [5]. Patient B35 presented with an intracerebral and leptomeningeal relapse between the fourth and fifth cycles of first-line consolidation therapy. Patient B38 developed a second intracerebral relapse during follow-up after second-line therapy. Monitoring of blood-based cfDNA concentration was unsuitable to detect disease recurrence beyond the blood–brain barrier in both patients (Figure 5C, upper right and lower left panels). Patient B50 was diagnosed with a local osteomedullary relapse directly following first-line consolidation therapy. At the time of relapse on day 349, the blood-based cfDNA concentration had increased from 2.59 ng/mL on day 180 and 1.45 ng/mL on day 247 to 4.22 ng/mL, thus correlating with disease recurrence (Figure 5C, lower right panel). We conclude that a favorable response to induction therapy triggers a strong reduction of initially high blood-based cfDNA levels, but that disease recurrence is not always detectable by monitoring blood-based cfDNA concentrations, particularly if the relapse occurs in the central nervous system.

### 3.4. Peripheral Blood-Derived cfDNA Is Suitable for Molecular Neuroblastoma Profiling in Infants

**Figure 6:**
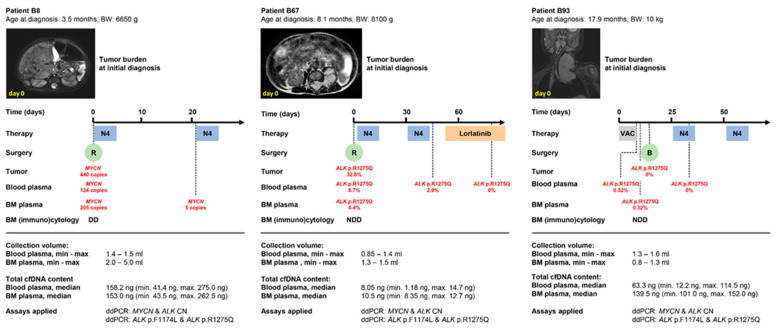
Overview of molecular ctDNA profiling results in 3 selected infants <18 months with neuroblastoma. Shown is the timeline from diagnosis through therapeutic interventions including drug therapies and surgery. Selected images reflect the tumor burden at initial diagnosis for patients B8 (**left panel**), B67 (**middle panel**) and B93 (**right panel**). Time points of bone marrow assessment by (immuno)cytology and droplet digital PCR (ddPCR) are summarized below the timelines. Sample volumes and cfDNA content available for ddPCR are also indicated. N4, chemotherapy regimen according to the NB2016 registry: doxorubicin, vincristine, cyclophosphamide; VAC, chemotherapy regimen according to the Cooperative Soft Tissue Sarcoma Study Group of the German Society of Pediatric Hematology and Oncology (GPOH): vincristine, actinomycin-D, cyclophosphamide. B, biopsy; BM, bone marrow; BW, body weight; R, resection; DD, disease detectable; NDD, no disease detectable.

While spontaneous cancer regression has been documented in different cancer types, this phenomenon is most prevalent with neuroblastoma [46]. Regression in the absence of tumor-specific therapy is observed in children up to 18 months with virtually any stage of disease if the tumor displays biologically favorable molecular characteristics. Localized disease progression, threatening symptoms, or tumors with biologically unfavorable molecular characteristics require therapeutic intervention in this very young age group. To test whether the very low blood and bone marrow volumes available from infants are sufficient to perform cfDNA-based molecular neuroblastoma profiling, we exemplarily employed our established multiplex ddPCR protocols [14,17] to monitor *MYCN* and *ALK* status in three patients with body weights ≤ 10 kg and ages at diagnosis below 18 months. Patient B8 was diagnosed with stage M neuroblastoma at the age and body weight of 3.5 months and 6650 g, respectively. Baseline imaging at initial diagnosis revealed bilateral adrenal neuroblastoma with loco-regional and distant metastases to the liver, skin, bone, and kidney (Figure 6, left panel). Molecular tumor tissue analysis identified a high-level *MYCN* amplification (Figure 6, left panel). Analysis of blood-derived and bone marrow-derived cfDNA detected *MYCN* copy numbers consistent with amplified *MYCN* at initial diagnosis and an *MYCN* gain on day 22, the latter pointing towards therapy-mediated clearance of the molecular markers from the biofluids (Figure 6, left panel). A minimal blood plasma volume of 1.4 mL, which contained 41.1 ng of purified total cfDNA, and a minimal bone marrow plasma volume of 2.0 mL, which contained 43.5 ng of total purified cfDNA, were sufficient to perform this analysis (Figure 6, left panel). Patient B67 was diagnosed with a paravertebral stage L2 neuroblastoma at the age and body weight of 8.1 months and 8100 g, respectively. Intraspinal tumor growth caused elongated compression of the spinal cord (Figure 6, middle panel) that required emergency neurosurgical intervention and systemic chemotherapy for low-risk disease (according to NB2016 Registry). Molecular analysis of tumor tissue identified an *ALK* p.R1275Q mutation with a MAF of 32.8% (Figure 6, middle panel). Longitudinal analysis of blood-derived cfDNA detected an *ALK* p.R1275Q mutation with 8.7% MAF at initial diagnosis and 2.9% MAF on day 38 (Figure 6, middle panel). The *ALK* p.R1275Q mutation was undetectable in blood-derived cfDNA on day 86, when conventional clinical response assessment judged that complete remission was achieved (Figure 6, middle panel). The *ALK* p.R1275Q mutation was detected at 4.4% MAF in bone marrow-derived cfDNA sampled at initial diagnosis, and no disease was detectable in standard bone marrow diagnostics (Figure 6, middle panel). A minimal blood plasma volume of 0.85 mL, which contained 1.18 ng of purified total cfDNA, and a minimal bone marrow plasma volume of 1.5 mL, containing 8.35 ng total purified cfDNA, were sufficient to perform this analysis (Figure 6, middle panel). The *ALK* p.R1275Q mutation not only served as a molecular marker for therapy response assessment and minimal residual disease monitoring in patient B67 but was also exploited as a druggable target, since additional chemotherapy cycles were substituted by treatment with the ALK-inhibitor, lorlatinib, in compassionate use (Figure 6, middle panel). Patient B93 was diagnosed with a cervical stage L2 neuroblastoma at the age and body weight of 17.9 months and 10 kg, respectively. 

**Figure 7:**
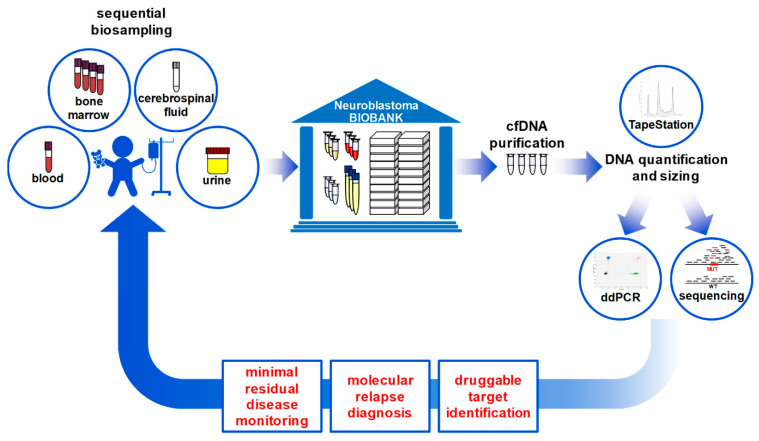
Graphical abstract demonstrating the workflow of liquid biopsy-based cfDNA diagnostics in patients with neuroblastoma.

Almost complete tracheal compression (Figure 6, right panel) required the emergency application of a systemic chemotherapy block in the absence of a histologically confirmed diagnosis followed by additional cycles of chemotherapy for low-risk disease, in line with NB2016 Registry guidelines. Molecular tumor tissue analysis detected neither *MYCN* nor *ALK* copy-number alterations nor *ALK* hotspot mutations (Figure 6, right panel). Analysis of blood- and bone marrow-derived cfDNA collected on days 3 and 6 of chemotherapy detected an *ALK* p.R1275Q mutation at 0.32% MAF (Figure 6, right panel). The data indicate that cfDNA analysis detected *ALK* p.R1275Q-mutated tumor clones or subclones that were not reflected in the tumor biopsy used to characterize molecular disease. No disease was detectable in standard bone marrow diagnostics, implying the high sensitivity ddPCR-based diagnostics using bone marrow-derived cfDNA. The *ALK* p.R1275Q mutation was undetectable in blood-derived cfDNA collected on day 31 (Figure 6, right panel), again indicating the rapid clearance of molecular markers from the bloodstream after therapy response. A minimal blood plasma volume of 1.3 mL, which contained 12.2 ng purified total cfDNA, and a minimal bone marrow plasma volume of 0.8 mL, containing 101.0 ng total purified cfDNA, were sufficient to perform this analysis (Figure 6, right panel). We conclude that absolute cfDNA amounts as low as 8 ng are sufficient to longitudinally monitor patient-specific tumor markers in infants with neuroblastoma, thus providing proof of concept that liquid biopsy-based circulating tumor DNA diagnostics can also contribute to disease monitoring and druggable target identification in infants diagnosed with neuroblastoma (graphical abstract provided in Figure 7).

## 4. Discussion

This study provides molecular-based guidelines to support biofluid sample biobanking from infants, children, and adolescents with neuroblastoma. Biofluid biobanking is minimally invasive and supports longitudinal sample collection at multiple time points during therapy and follow-up to sustainably expand current exclusively tissue-based biobanking and molecular pathological disease profiling. The DNA size distribution and cfDNA concentrations reported here for more than 600 biofluid samples collected at a single center under standard operating procedures reflecting international experience [37] provide the molecular basis for decision-making about how much sample volume to store for efficient down-stream analysis. The “the more the better” principle is opposed to the low body weight of infants and children, requiring careful technical and regulatory considerations. While the 7.5 mL blood volume routinely collected from adult patients with different cancer entities [47] is not applicable in the pediatric population, the recommendations provided here (1 mL of blood and bone marrow plasma, 2 mL of CSF, complete urine portions) reflect not only molecular requirements but are fully in line with clinical feasibility.

Schmelz et al. reported high spatial and temporal heterogeneity in somatic mutations and copy-number alterations that were mirrored on the transcriptomic level in a transcriptomic and genomic profiling study of multiregional biopsies from 10 patients with neuroblastoma [48]. We recently investigated whether cfDNA-based diagnostics can detect intratumor heterogeneity not reflected in single neuroblastoma biopsies by comparing marker profiles in matched blood and tumor tissue samples [21]. Our findings supported that neuroblastoma is spatially genetically heterogeneous and that this clonal heterogeneity is reflected in circulating cfDNA [21]. This finding has direct implications for ALK-inhibitor therapy selection [49], so far the most promising targeted therapy for approximately 7% of neuroblastomas that are driven by activating *ALK* mutations [50]. Altogether, these studies underline the urgent need to provide molecular profiling of the dynamic changes in the molecular landscape of neuroblastoma caused by endogenous competition of genetically divergent tumor cell clones and the exogenous pressure exerted on tumor cells by multimodal cancer therapy. Such comprehensive analyses require sophisticated liquid biopsy biobanking approaches, as outlined in this study.

Characterization of DNA size distribution in blood plasma underlines the importance to assess every single sample. In line with previous reports [18], approximately 24% of samples did not contain primarily cfDNA, despite the exclusive use of EDTA blood collection tubes and a sample processing time of 1 h. Neither a reddish appearance of the blood plasma indicative of red blood cell lysis nor a cloudy appearance of the blood plasma pointing towards elevated blood lipid levels correlated with poorer cfDNA quality, suggesting that such samples should not automatically be excluded. DNA purified from bone marrow plasma differs from the profile found in blood plasma. The overall higher total DNA content may reflect the high cellular content in the bone marrow niche recently shown to comprise as many as 45 different cell types [51]. The proportion of samples containing primarily cfDNA was larger among patients with neuroblastoma compared to healthy individuals. The potential of bone marrow plasma-based cfDNA profiling for minimal residual disease monitoring warrants further investigation. The absolute quantities of cfDNA in cerebrospinal fluid are as low as reported for patients with primary CNS tumors [52], but sufficient to serially assess minimal residual disease in patients with high-risk neuroblastoma and central nervous system metastases [21,53]. Only low circulating cell-free tumor DNA levels circulate in blood from these individuals, possibly due to the blood–brain barrier [25]. The studies employed low-coverage whole-genome sequencing, digital PCR, and targeted amplicon sequencing, altogether demonstrating that both targeted and untargeted approaches can be employed to decode the molecular information embedded in cerebrospinal fluid [21,25,52,53]. The least invasively collected biosample, urine, contains similarly low amounts of cfDNA as CSF. Urine-based DNA fragment size spans a broad spectrum between 100 and 1000 bp, leading to a characteristic broad peak in the electropherogram [54]. Urine from patients with neuroblastoma contains higher amounts of DNA spanning a broad spectrum of fragment lengths compared to urine collected from pediatric controls. This observation based on a comparatively small sample number deserves further investigation.

Systemic inflammatory response syndrome and infection trigger an increased release of cfDNA from various organs through apoptosis and necrosis [5]. These conditions may contribute to the undulating blood-based cfDNA concentrations observed during longitudinal monitoring in individual patients. The correlation between total cfDNA concentration and disease activity has been extensively studied by Sue et al. [29,30] and was partly confirmed in our study. Total cell-free DNA content may be suitable for integration into a panel containing additional specific markers for disease activity. Altogether, specific markers for disease activity are expected to be most sensitive for disease monitoring.

Cell-free DNA studies in patients with neuroblastoma have mostly focused on high-risk disease, for which the need for improved diagnostics and therapies is most urgent [5,6]. We demonstrate that the promise of liquid biopsies is not restricted to these cases. The small volumes of blood plasma available from infants with localized disease are sufficient to detect and monitor neuroblastoma-specific markers. While this study exclusively focused on cfDNA, several other tumor surrogates in liquid biopsies such as cfRNA, proteins, metabolites, extracellular vesicles, and circulating tumor cells are gaining increasing interest [5,6]. Some of these tumor surrogates require specific and immediate sample processing prior to first cryopreservation, adding an additional layer of complexity to successful liquid biopsy biobanking.

## 5. Conclusions

Biofluids are a less invasive source of biomarkers to monitor cancer in patients and support therapeutic decision-making than surgically acquired tumor tissue. Collection and biobanking of biofluids require exact knowledge of tumor surrogate content. Otherwise, elaborately collected samples may not be banked in sufficient volumes to be suitable for downstream analysis. Based on the analysis of 600 DNA electropherograms of biofluid samples, we report recommendations for the sampling of blood plasma, bone marrow plasma, cerebrospinal fluid, and urine from patients with neuroblastoma for cfDNA-based studies. The recommendations provided here (1 mL of blood and bone marrow plasma, 2 mL of CSF, complete urine portions) reflect not only molecular requirements but are fully in line with clinical feasibility. Optimal liquid biopsy biobanking is an indispensable step before the power of liquid biopsies can be wielded to revolutionize clinical care for infants and children with neuroblastoma in prospective studies accompanying multi-center clinical trials.

## Data Availability

Original TapeStation electropherogram and droplet digital PCR data presented in this study are available on request from the corresponding author.

## Supplementary Materials

The following supporting information can be downloaded at: https://www.mdpi.com/article/10.3390/cancers14092080/s1, Figure S1: Influence of sample processing time on blood-based DNA characteristics; Table S1: Patient and tumor characteristics in the high-risk neuroblastoma study subpopulation.
