# Supplementary material for: Circulating Cell-Free DNA Assessment in Biofluids from Children with Neuroblastoma Demonstrates Feasibility and Potential for Minimally Invasive Molecular Diagnostics
Source: Cancers (Basel). 2022 Apr 21;14(9):2080. doi: 10.3390/cancers14092080 (PMC9099910; doi:10.3390/cancers14092080)
Supplement: Supplementary file 1 [file cancers-14-02080-s001.zip › cancers-1666838-supplementary.pdf]

SUPPLEMENTARY DATA

Circulating Cell-Free DNA Assessment in Biofluids from Children with Neuroblastoma Demonstrates Feasibility and Potential for Minimally Invasive Molecular Diagnostics

Marco Lodrini, Jasmin Wünschel, Theresa M. Thole-Kliesch, Maddalena Grimaldi, Annika Sprüssel, Rasmus B. Linke, Jan F. Hollander, Daniela Tiburtius, Annette Künkele, Johannes H. Schulte, Erwin Lankes, Thomas Elgeti, Patrick Hundsdörfer, Kathy Astrahantseff, Thorsten Simon, Angelika Eggert and Hedwig E. Deubzer

SUPPLEMENTARY TABLES

Table S1. Patient and tumor characteristics in the high-risk neuroblastoma study subpopulation.

Factor	No. of patients	% of study population
	Sex	
male	32	64
female	18	36
	Age at diagnosis	
< 18 months	14	28
≥ 18 months	36	72
	MYCN status[1]	
diploid	25	50
amplified	25	50
	1p36 status[2]	
no aberration	23	46
aberration	24	48
not available	3	6
	ALK status[3]	
wildtype	20	40
gain	2	4
mutation	15	30
not available	13	26
	Overall survival status	
alive	33	66
succumbed to disease	15	30
died of other causes	2	4

[1] Genomic copies of the *MYCN* oncogene as analyzed by fluorescence in situ hybridization, Southern blot and hybrid capture-based panel sequencing. *MYCN* amplification was defined as >8 genomic copies. [2] Genomic status of the chromosome 1p36 region as analyzed by fluorescence in situ hybridization and PCR. [3] *ALK* status as analyzed by hybrid capture-based panel sequencing.

SUPPLEMENTARY FIGURES

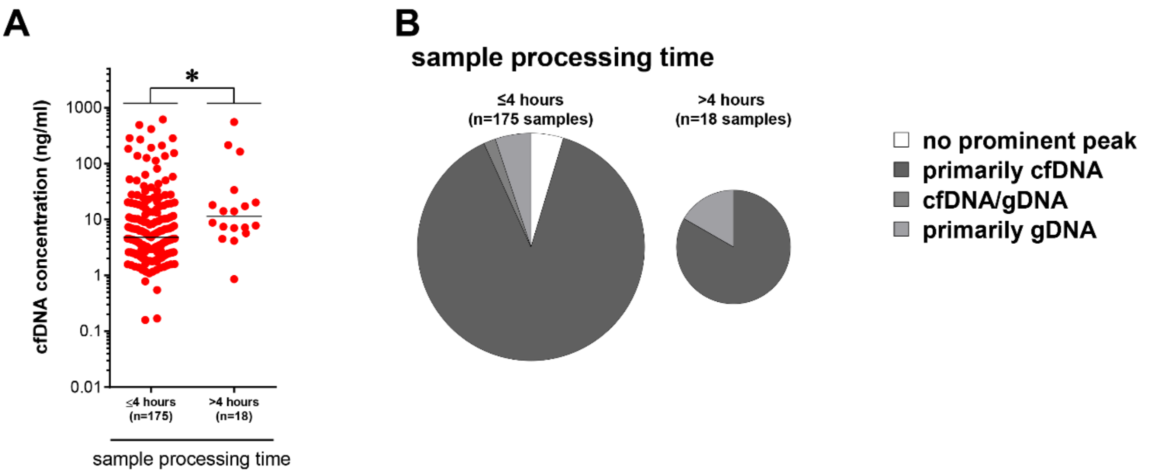

Figure S1. Influence of sample processing time on blood-based DNA characteristics. (A) DNA extracted from blood plasma was analyzed using the cell-free DNA ScreenTape assay (Agilent) and the Agilent 4200 TapeStation System. Each symbol depicts an individual measurement. Data are categorized into two different subgroups dependent of the sample processing time. *$P < 0.05$. (B) Piecharts demonstrating the distribution of electropherogram profiles characterized by (i) no prominent peak, (ii) primarily cfDNA, (iii) similar amounts of cfDNA and high molecular weight genomic DNA and (iv) primarily high molecular weight genomic DNA. Data are categorized into two different subgroups dependent of the sample processing time.
